# Developmental Toxicity of Photolithography-Relevant Per- and Polyfluoroalkyl Substances (PFAS) Reveals Concerns for Less-Studied Functional Groups

**DOI:** 10.1021/acs.est.5c09577

**Published:** 2025-12-24

**Authors:** Yuexin Cao, Hajar Smaili, Hazel Q. Shanks, Brooke E. Tvermoes, Shan Niu, Ruiwen Chen, Neil A. Hukriede, Carla A. Ng

**Affiliations:** † Department of Civil & Environmental Engineering, 6614 University of Pittsburgh, Pittsburgh, Pennsylvania 15261, United States; ‡ Center for Integrative Organ Systems, Department of Cell Biology, University of Pittsburgh, Pittsburgh, Pennsylvania 15213, United States; § Chief Sustainability Office, 3261 IBM, Durham, North Carolina 27709, United States; ∥ Advanced Interdisciplinary Institute of Environment and Ecology, Beijing Normal University, Zhuhai, Guangdong 519087, China; ⊥ Department of Environmental and Occupational Health, University of Pittsburgh, Pittsburgh, Pennsylvania 15261, United States

**Keywords:** PFAS hazard assessment, semiconductor industry, zebrafish embryo assay, lethal concentration, transcriptional analysis, modes and mechanisms of action, lipid metabolism

## Abstract

Per- and polyfluoroalkyl substances (PFAS) are used throughout semiconductor manufacturing, including photolithography, yet many remain toxicologically uncharacterized. This study used the zebrafish embryo assay to assess developmental toxicity and gene expression changes (*fgf10a*, *igf1*, *fabp10a*, *pparg*, and *mttp*) linked to growth and lipid metabolism for nine legacy and emerging photolithography-relevant PFAS. Exposure induced increased lethality and common malformations, including failure of swim bladder inflation and yolk sac edema. Transcriptional analysis revealed that certain PFAS, especially triphenylsulfonium perfluoro-1-butanesulfonate, 1H,1H,2H,2H-perfluorooctane sulfonic acid, perfluorooctanoic acid, and bis­(1,1,2,2,3,3,4,4,4-nonafluoro-1-butanesulfonyl)­imid, significantly downregulated *pparg* and *mttp* expression, even at environmentally relevant concentrations. Our findings raise concerns that certain PFAS actively used in photolithography, including some with less-studied functional groups (e.g., perfluoroalkyl dicarboxylic acids and perfluorosulfonamides), are more toxic than equivalent-chain monocarboxylic acid and legacy PFAS, respectively, providing mechanistic insight into PFAS-induced toxicity. These results help inform the selection of safer chemicals in semiconductor manufacturing.

## Graphical abstract

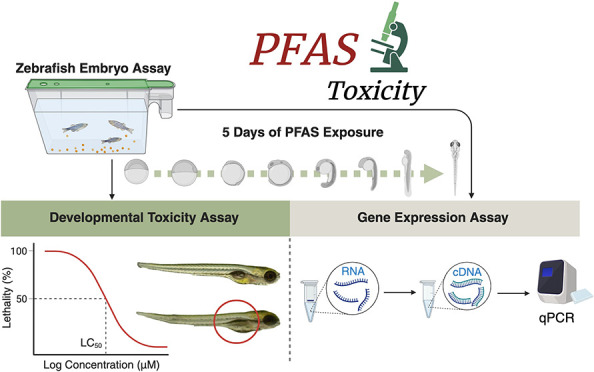

## Introduction

As the global semiconductor industry is undergoing a paradigm shift, driven by the rise of artificial intelligence, quantum computing, and next-generation automotive technologies, the demand for high-performance, energy-efficient microelectronics is unprecedented.^1^ According to a 2024 McKinsey report, the global semiconductor market reached $600 billion in 2021 and is projected to exceed $1 trillion by 2030.^2^ However, sustaining and expanding semiconductor innovation requires overcoming critical challenges in materials engineering, heterogeneous integration, and environmental sustainability. From an environmental perspective, the use of per- and polyfluoroalkyl substances (PFAS) is one of the greatest challenges for modern industry.^1^

PFAS are a class of synthetic fluorinated chemicals that have been widely used in various industries, including in photolithography for semiconductor manufacturing.^3^ Photolithography, a critical process in semiconductor production, involves transferring intricate patterns onto a photoresist-coated substrate.^4^ In this process, many PFAS-containing chemicals are employed, such as triphenylsulfonium perfluoro-1-butanesulfonate (TPS-PFBS) in photoacid generators (PAGs), perfluoropentanoic acid (PFPeA) in antireflective coatings, and 1H,1H,2H,2H-perfluorooctane sulfonic acid (6:2 FTS) in etching agents.^3^ Safer alternatives that meet the multifaceted performance requirements necessitated by many uses of PFAS in photolithography processes do not currently exist.^5^

There are several key use applications in which PFAS are used in semiconductor photolithography.^3,6^ Recent work by the Semiconductor PFAS Consortium has developed environmental release pathways from photolithographic processes identifying potential routes for PFAS releases.^7^ Long-chain legacy and emerging PFAS have been detected in wastewater from semiconductor fabrication plants and electronics fabrication facilities (fabs),^8,9^ as well as in rivers receiving fab discharges and surface waters surrounding these sites.^10,11^ While long-chain legacy perfluorinated alkyl acids are known to bioaccumulate and induce effects including reproductive and developmental toxicity, endocrine disruption, neurotoxicity, and tumor induction,^12^ many of the PFAS emerging from electronics manufacturing are poorly characterized.^13^ Therefore, there is a critical need to understand the toxicity and mechanisms of action of other PFAS that are currently important for industrial uses, including photolithography, to better design safer chemistries.

The zebrafish is a widely used vertebrate model for evaluating the adverse effects of xenobiotics because of its small size, rapid development, high fertility, and genetic similarity to humans.^14,15^ The zebrafish embryo assay is considered an alternative to traditional adult vertebrate animal models because of the short assay duration, which typically requires 96–144 h of exposure, the ability to visualize adverse effects early during larval development, and characteristics meeting the 3R principles (replacement, reduction, and refinement).^16^ Previous studies have used embryonic zebrafish to test the developmental toxicity and behavioral effects of C4 to C8 perfluoroalkyl carboxylic acids (PFCAs), C4, C6, and C8 perfluoroalkyl sulfonic acids (PFSAs),^17–20^ and some emerging PFAS.^17^

In this study, we selected nine structurally diverse PFAS (Table 1) in consultation with semiconductor industry partners and recent surveys of photolithography chemicals.^8,21^ To ensure representative coverage of photolithography-relevant classes and to enable comparison of well-studied and less-studied PFAS, we included legacy compounds (PFOS and PFOA) detected in fab wastewater and emerging PFAS with diverse functional groups currently used in photolithography that are relatively less-studied (e.g., perfluoroalkyl sulfonamides, short-chain acids, and a dicarboxylic acid). We evaluated their developmental toxicity via the zebrafish embryo assay and performed transcriptional analyses on zebrafish larvae exposed to environmentally relevant PFAS concentrations to investigate their toxicological mechanisms and potential low-dose effects that are not observable in morphological assays. Our findings will provide valuable insights into the safety of PFAS used in photolithography, contributing to a better understanding of their modes and mechanisms of action and the resulting environmental and biological impacts.

**Table 1:**
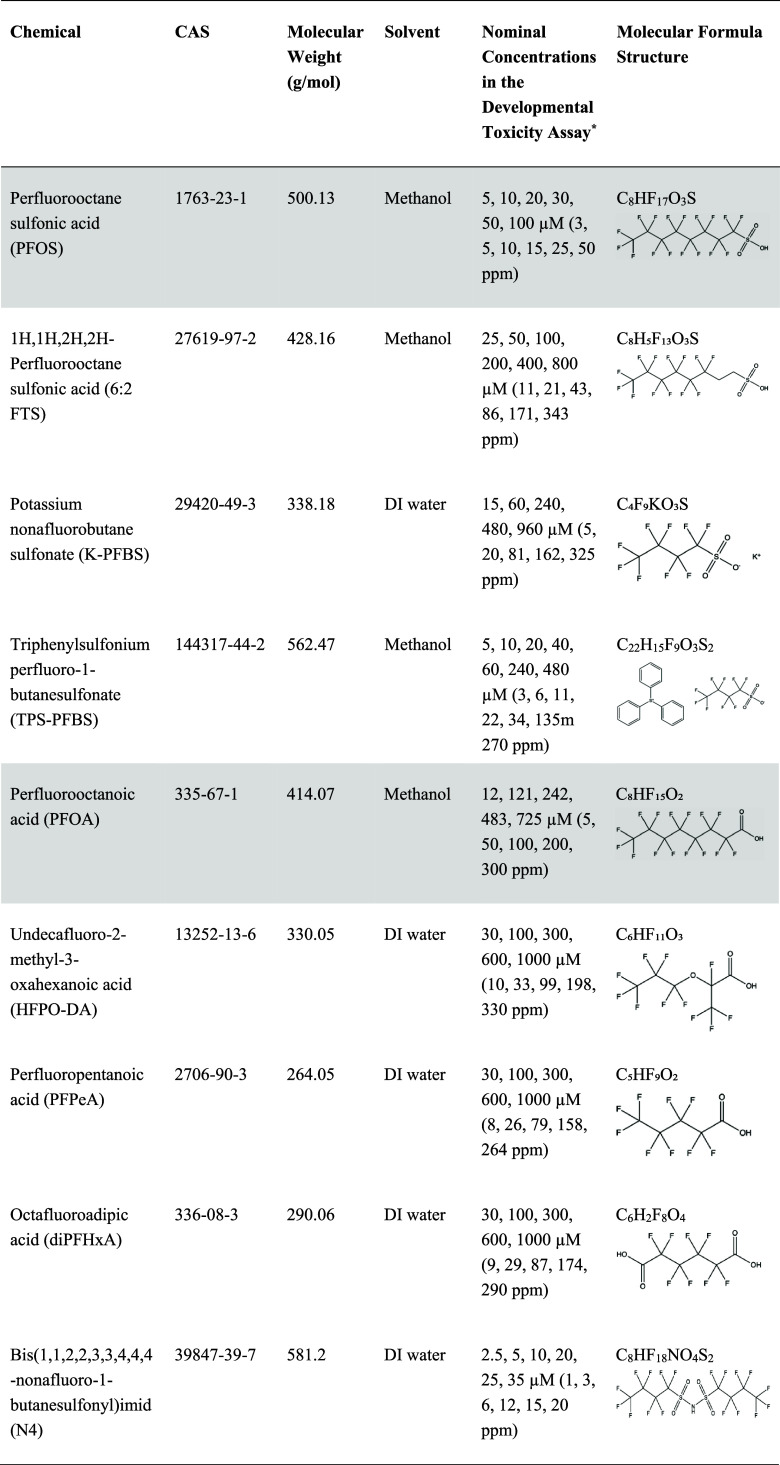
Information, Structures, and Nominal Concentrations of Tested PFAS in the Zebrafish Embryo Assay^a^

a *Concentrations in parentheses are in parts per million (ppm) units, provided for convenience. Nominal concentrations were confirmed by LC-MS/MS. Shaded cells indicate legacy PFAS that have been phased out in the United States.^3^ Concentrations for each PFAS in the developmental toxicity assay were determined based on initial dose range-finding experiments in the pilot study.

## Materials and Methods

### Zebrafish Maintenance

All procedures involving zebrafish followed the University of Pittsburgh guidelines and standards, and a protocol (protocol number: 19116421) was approved by the Institutional Animal Care and Use Committee. The zebrafish embryo assay was conducted and modified in accordance with the OECD Test Guideline (No. 236, Fish Embryo Toxicity Test)^22^ as described in sections that follow. Embryos were collected from wild-type (AB strain) adult zebrafish (*Danio rerio*). Details of husbandry and embryo collection can be found in SI Section S1.1. The collected embryos were gently rinsed in clean E2 media (a buffered saline solution for raising embryonic zebrafish) and stored in the incubator at 28.0 °C until 6 h post fertilization (hpf). Embryos remained with the chorion intact until hatching naturally occurred for all subsequent exposures.

### Chemical Preparation

PFAS used in this study (names and CAS numbers noted in Table 1) were purchased from Synquest Laboratories, with the exception of TPS-PFBS, which was purchased from Sigma-Aldrich, and bis­(1,1,2,2,3,3,4,4,4-nonafluoro-1-butanesulfonyl)­imid (N4), which was provided by our industrial liaisons from EMD. Methanol (HPLC grade) and ammonium acetate (Crystalline/Certified ACS) were obtained from Fisher.

Each PFAS was tested alongside a negative control (NC) group, a positive control (PC) group, and the treatment groups at various concentrations. The NC was zebrafish embryo E2 media water or E2 media water with 0.5% methanol added, depending on whether methanol was used as the solvent for PFAS preparation (Table 1). E2 media water is composed of 15 mM NaCl, 0.5 mM KCl, 1.0 mM MgSO_4_, 150 μM KH_2_PO_4_, 50 μM Na_2_HPO_4_, 1.0 mM CaCl_2_, and 0.7 mM NaHCO_3_ and provides an important salt balance for zebrafish embryo development. Methanol was added as a solvent to aid in solubilizing substances with low water solubility, and 0.5% was determined by Rericha et al.^20^ as the highest concentration that will not cause side effects on embryos. The PC was 100 μM PFOS, selected because it always achieved more than 90% larval death at the end of the exposure in our multiple pilot experiments. For each chemical, we tested 5–7 different concentrations as treatment groups in the developmental toxicity assay (Table 1) and two concentrations in the gene expression assay (listed in the chemical exposure section).

Prior to exposure, the concentrations of the PFAS stock solutions were validated using ultrahigh-performance liquid chromatography coupled with a triple quadrupole mass spectrometer (UHPLC-MS/MS; Vanquish Flex and TSQ Quantis, Thermo Scientific). Details of the instrumental analysis are described in SI Section S1.2. To avoid blank contamination, materials used in the chemical exposure experiments, including exposure plates, sample collection tubes, and solvents (i.e., E2 media and methanol), were tested and confirmed to have undetectable PFAS levels prior to use. In addition, pH values in the treatment group were measured and adjusted as needed to remain within the range of 6.5–8.5 to prevent adverse effects on zebrafish development.^22^

Given that PFAS are surface-active, we measured the surface tension of all PFAS solutions at various concentrations to help explain any potential effects of surface tension on zebrafish larvae during exposure. Surface tension was analyzed using the Young–Laplace method with a Biolin Scientific Attension Theta optical tensiometer. The resulting surface tension values are presented in Section S1.2 of the SI, Figure S1.

### Chemical Exposure

In the developmental toxicity assay, from 6 hpf, each individual embryo was placed into a single well of, first, a 96-well mesh plate (Millipore Sigma, Catalog Number: MANMN4010), which contained chemicals identical to those in the exposure plate. This step served as a pretreatment to prevent the embryonic medium water present in the embryo transfer process and on the embryo surface from diluting the exposure solution. Each well of the mesh plate contained 400 μL of a working solution. After pretreatment, embryos were transferred to the 96-well exposure plate (Millipore Sigma, Catalog Number: MAMCS9610) via the mesh insert. Each well of the 96-well plate contained 250 μL of working solution. Each treatment and control group had three replicates of 16 embryos, resulting in 48 embryos per group.

In the gene expression assay, 16 zebrafish embryos were placed into each well of a six-well plate starting from 6 hpf, with three wells used per concentration as triplicates. Dead embryos were removed during exposure to maintain optimal conditions for surviving embryos. Two concentrations of each PFAS were tested: a low dose based on the highest concentration of PFBS detected in electronics fabrication facility wastewater (8040 ng/L, equivalent to 0.03 μM) as reported by Jacob et al.,^8^ representing a worst-case environmental scenario, and a high dose based on the highest concentration in the developmental toxicity assay without significant lethality but where malformations were observed. The high dose for each PFAS was as follows: PFOS (2.5 μM), 6:2 FTS (50 μM), K-PFBS (600 μM), TPS-PFBS (60 μM), PFOA (100 μM) HFPO-DA (30 μM), diPFHxA (30 μM), PFPeA (30 μM), and N4 (2.5 μM).

After exposure, the plates were sealed using a sealing film (Bio-Rad, Catalog Number: MSA5001) and a lid to limit evaporation, and placed into an incubator at 28.0 °C. The exposure media was refreshed every day from 1 to 4 days post fertilization (dpf), maintaining an approximately constant exposure concentration throughout the duration of the experiment.

To ensure high-quality data and prevent the generation of false positive or negative results, strict adherence to quality assurance and quality control measures is essential. Alongside following the OECD Test Guideline No. 236 (Fish Embryo Toxicity Test)^22^ to meet test validity criteria, we emphasize the need for international standardization of the zebrafish embryo assay. One of the biggest challenges of modern toxicology is developing consistent testing protocols and reaching global agreement on their use.^23^ For the zebrafish embryo assay, specific aspects in protocol design, such as initial exposure time, exposure duration, use of embryos with or without an intact chorion, and static versus media replacement methods, should be consistently standardized to enhance reproducibility and comparability across studies.

### Developmental Toxicity Assay

Hatching status, lethality, and malformations were assessed in the developmental toxicity assay. Hatching typically happens between 48 and 72 hpf.^24^ The rate of delayed hatching was determined at 72 hpf. At the end of the exposure (120 hpf), two independent observers scored zebrafish larvae for lethality and several sublethal end points. Coagulation of embryos, lack of somite formation, nondetachment of the tail, nonhatching, and lack of heartbeat were used to determine lethality. Live larvae exhibiting a weak and slower heartbeat, insensitivity to external stimuli, and/or abnormal buoyancy control were labeled as “weak”. This categorization was used to identify the difference between healthy live larvae and abnormal larvae with a higher risk of mortality, independent of other scored malformations. Sublethal end points evaluated were curved body axis, failed swim bladder inflation, yolk sac edema, pericardial edema, short trunk, and bent tail. When there were discrepancies in the assessments between the two observers, a reevaluation was conducted to ensure accuracy and consistency.

### Gene Expression Assay (qPCR)

At the end of the exposure, 16 anesthetized 120-hpf zebrafish larvae from each well were collected into a 1.5 mL Eppendorf Biopur safe-lock microcentrifuge tube, with all exposure medium removed. A total of 900 μL of QIAzol reagent (QIAGEN) was added to each tube, and the samples were incubated at room temperature for 15 min to lyse the tissues and release RNA. The larvae were homogenized by pipetting until fully dissolved, then flash-frozen, and stored at −80 °C for subsequent RNA extraction. Detailed procedures for RNA extraction, cDNA synthesis, primer validation, qPCR conditions, and gene expression analysis are provided in SI Section S1.3.

### Data Analysis

According to the OECD Test Guideline (No. 236, Fish Embryo Toxicity Test),^22^ a valid plate requires that the NC group has a survival rate of at least 90% by the end of the test and ≥80% of embryos hatched, while the PC group should induce at least 30% mortality. Plates failing these criteria were excluded. Delayed hatching, lethality, and malformation rates were calculated for each control and treatment group, with malformation rates defined as the proportion of malformed live larvae among total live larvae. Statistical differences between control and treatment groups were assessed using the Pearson chi-square test/Fisher exact test (IBM SPSS Statistics 29.0.2.0). Dead larvae were excluded from malformation analysis, resulting in variable missing data across the groups. Previous studies on statistical analysis have suggested that analyses with more than 10% missing data are prone to bias, while analyses with more than 40% missingness should be considered as hypothesis-generating rather than definitive.^25^ Therefore, red and blue stars were used to flag such cases, respectively, indicating potential limitations and the impacts of missing data on statistical conclusions.

Dose–response curves corresponding to lethality and malformation rates were created using GraphPad Prism V10.3.1 through nonlinear regression analysis employing a Hill slope curve. Lethal concentration 50% (LC_50_) values indicating the exposure concentration leading to 50% lethality and half-maximal effective concentration (EC_50_) values indicating the exposure concentration resulting in a 50% malformation rate for each specific sublethal end point were calculated based on the dose–response curve.

To consider the overall developmental toxicity, we calculated the developmental toxicity score for each individual zebrafish larva based on a method adapted from Gaballah et al.^17^ The developmental toxicity scores of normal larvae, larvae exhibiting a single abnormality (including those labeled as “weak” and with malformations), larvae exhibiting ≥2 abnormalities and dead larvae were 0, 1, 2 and 3, respectively. The Pearson chi-square test/Fisher exact test (IBM SPSS Statistics 29.0.2.0) was performed to test for significant differences in developmental toxicity scores between treatment groups and the control group. All the data were reported as mean ± standard deviation (SD). *p*-values < 0.05 (*), < 0.01 (**) and < 0.001 (***) were considered as statistically significant.

In the gene expression assay, the relative expression ratio data were analyzed using GraphPad Prism V10.3.1 and presented as means ± SDs. Differences of the relative expression ratio between the control and PFAS groups were determined by a one-way analysis of variance (ANOVA) followed by Tukey’s multiple comparisons test. *p*-values < 0.05 (*), < 0.01 (**) and < 0.001 (***) were considered as statistically significant.

## Results

### N4, PFOS, and PFOA Exhibit the Highest Lethality (LC_50_) among the Tested PFAS

Zebrafish lethality was assessed on the basis of coagulation, absence of heartbeat, or nonhatching status (i.e., embryos showed early development but died inside of the chorion). Based on the tested concentrations, the LC_50_ values were determined only for PFOS, PFOA, and N4. Significant lethality was observed for PFOS at concentrations as low as 10 μM (*p* < 0.05), whereas N4 triggered similar effects starting at 25 μM (*p* < 0.05). PFOA exhibited lethality at a markedly higher threshold, with mortality increasing sharply from 242 μM (*p* < 0.001). In contrast, 6:2 FTS and K-PFBS demonstrated relatively lower lethality, with LC_50_ values exceeding 800 and 960 μM, respectively (Figure 1). Surface tension at the dosed concentrations was measured and is unlikely to be a factor in observed zebrafish lethality. Surface tension data and further discussion are in SI Figure S1 and Section S1.2.

**Figure 1:**
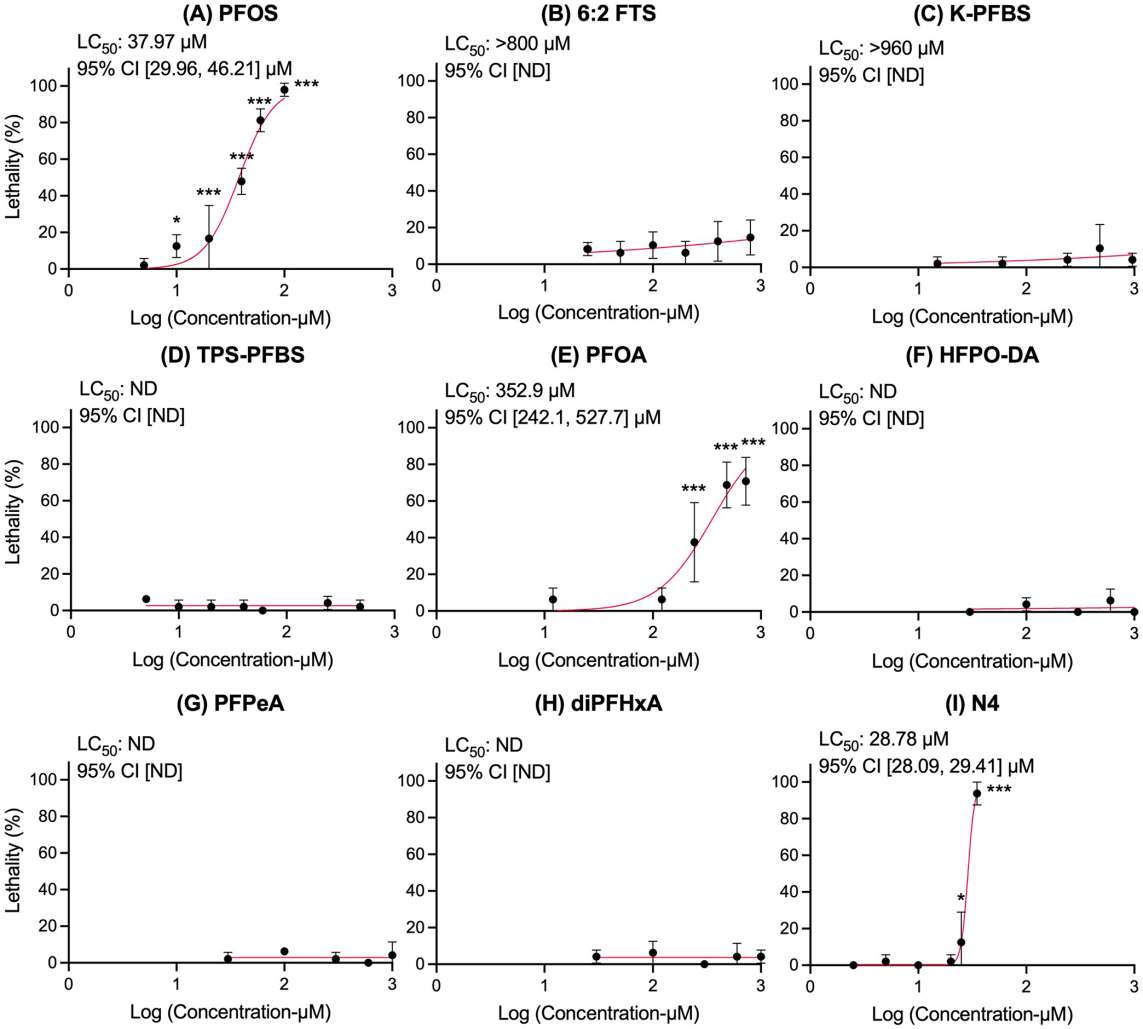
Dose–response curves for lethality in zebrafish embryos exposed to (A) PFOS, (B) 6:2 FTS, (C) K-PFBS, (D) TPS-PFBS, (E) PFOA, (F) HFPO-DA, (G) PFPeA, (H) diPFHxA, and (I) N4. **Notes**: statistical significance relative to the NC group was determined by the Pearson chi-square test/Fisher’s exact test (*p < 0.05; **p < 0.01; ***p < 0.001). “ND” indicates that the tested concentrations did not yield enough lethal outcomes to accurately determine the LC_50_ value or the 95% confidence interval (CI) could not be calculated due to high variability. Error bars indicate one SD from the mean.

The calculated LC_50_ values, in descending order of lethal potency, were as follows: N4 (28.78 μM), PFOS (37.97 μM), PFOA (352.90 μM), 6:2 FTS (>800 μM), and K-PFBS (>960 μM). No significant differences in mortality were observed between the negative control group and those exposed to 6:2 FTS, K-PFBS, TPS-PFBS, HFPO-DA, PFPeA, and diPFHxA.

### Failed Swim Bladder Inflation and Yolk Sac Edema Are Most Prevalent PFAS-Induced Malformations

Some larvae that lived during our assessment of lethality nevertheless exhibited signs of weakness when compared to controls. These “weak larvae” were observed across all nine PFAS treatments, with significantly greater occurrence in at least one treatment group for PFOS, 6:2 FTS, TPS-PFBS, PFOA, diPFHxA, and N4 (SI, Figure S2).

Failed swim bladder inflation and yolk sac edema were the most frequently observed sublethal malformations across all exposures. EC_50_ values for these end points were calculated based on dose–response curves (Figures 2B and 3C), although some data points were missing due to 100% lethality in high-concentration groups (e.g., 60 and 100 μM PFOS). According to the EC_50_ values, PFAS potency for failed swim bladder inflation was ranked as follows: PFOS (<5 μM) > N4 (10.78 μM) > PFOA (150.1 μM) > TPS-PFBS (1024 μM) > K-PFBS (1075 μM). For yolk sac edema: N4 (24.34 μM) > TPS-PFBS (465.2 μM) > PFOA (812 μM) > K-PFBS (1100 μM). However, EC_50_ values could not be determined for certain compounds, even at relatively high concentrations, due to high variability, limited response range, or the absence of clear concentration-dependent trends within the tested range (weak potency).

**Figure 2:**
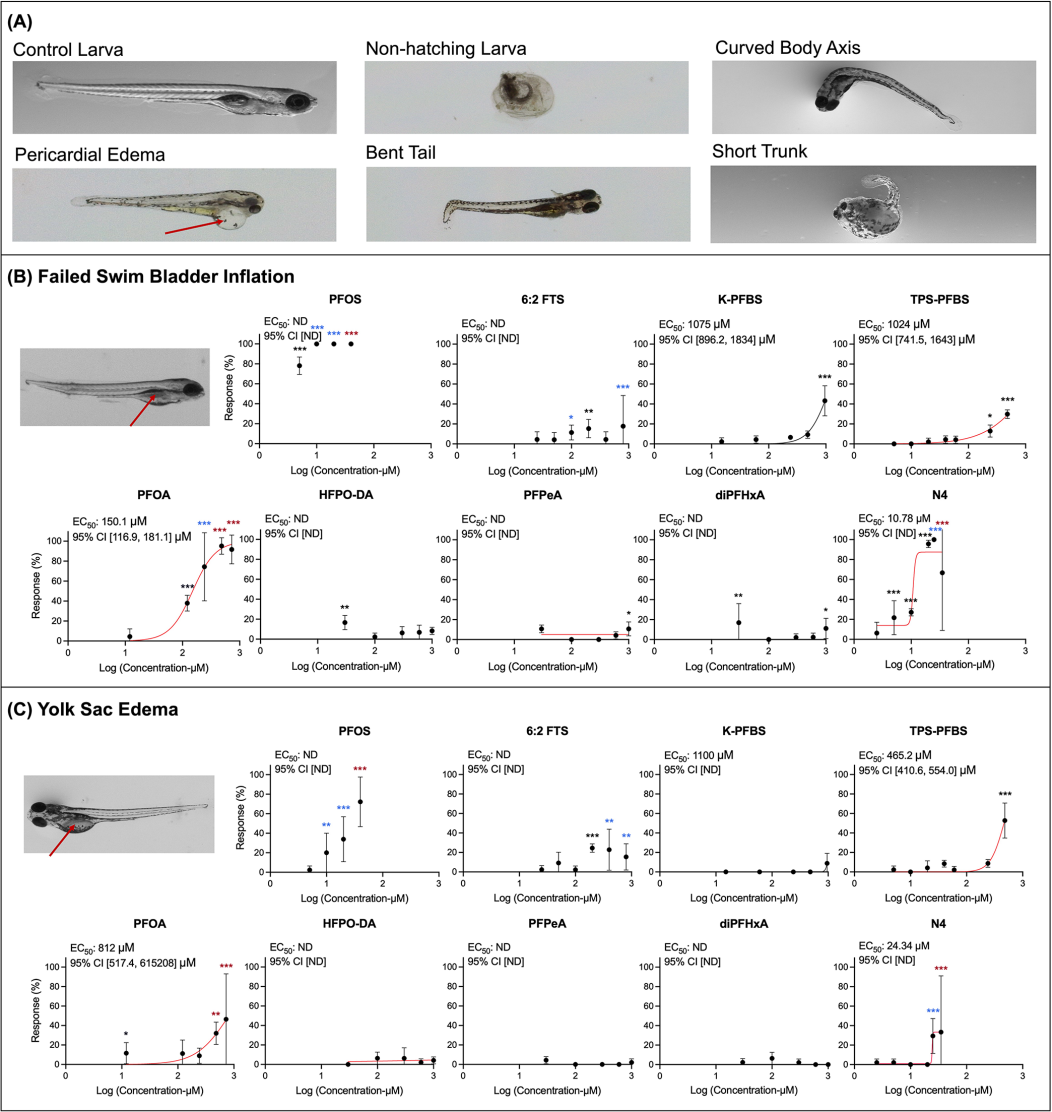
Representative images of (A) normal zebrafish larva and zebrafish larvae exhibiting malformations (red arrow); representative image and dose–response curves for (B) failed swim bladder inflation (red arrow), and (C) yolk sac edema (red arrow). **Notes**: dead larvae were not included in the calculation of the malformation rates. Statistical significance relative to the NC group in (B, C) was determined by the Pearson chi-square test/Fisher’s exact test (*p < 0.05; **p < 0.01; ***p < 0.001). Blue and red stars indicate that the statistical analysis result was from a set of data including more than 10 and 40% missing data, respectively. “ND” indicates that the tested concentrations did not result in a sufficient number of sublethal outcomes to determine the EC_50_ value accurately, or 95% CI data cannot be calculated based on the current data due to high variability. Error bar indicates one SD from the mean.

**Figure 3:**
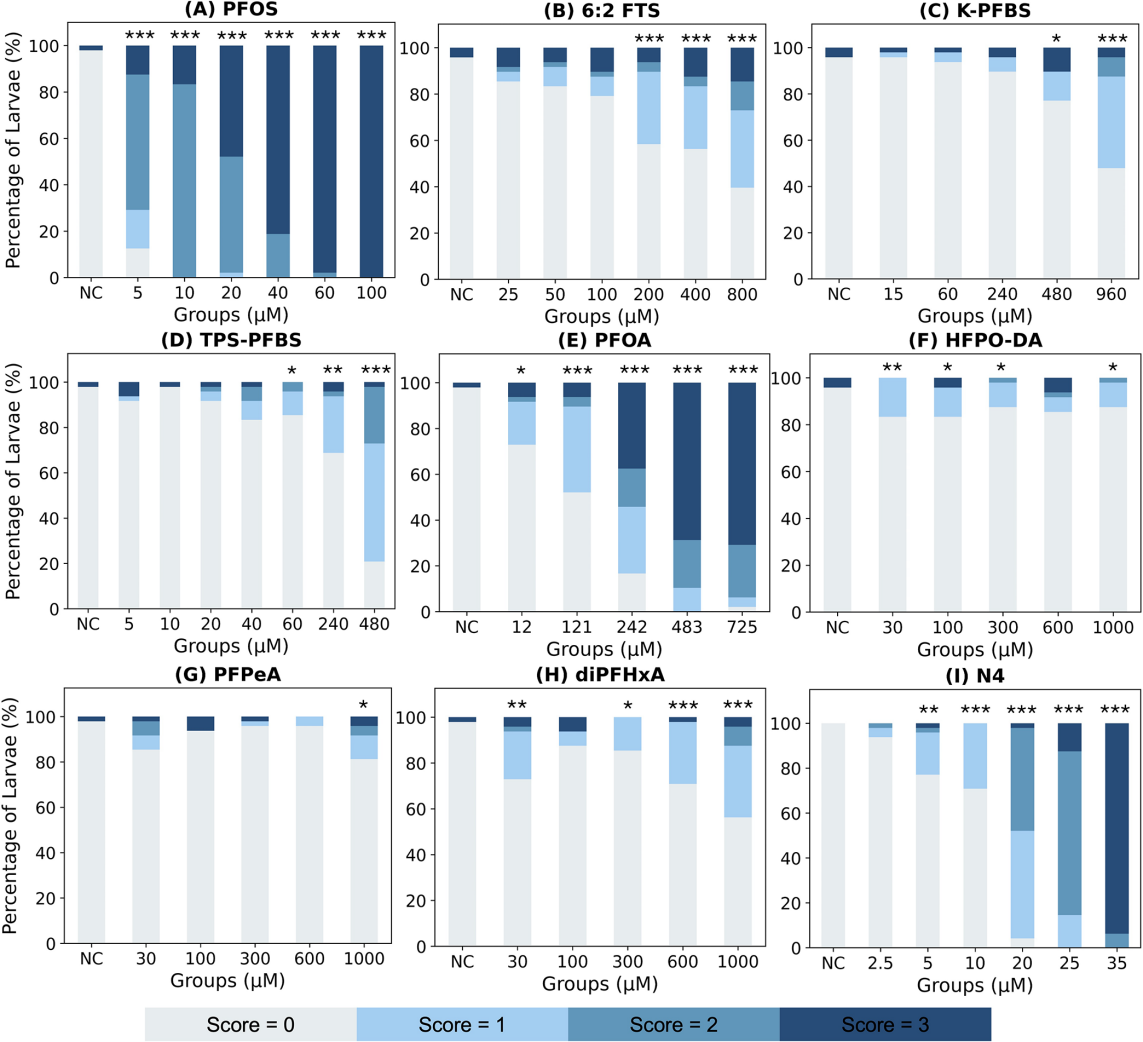
Developmental toxicity scores of (A) PFOS, (B) 6:2 FTS, (C) K-PFBS, (D) TPS-PFBS, (E) PFOA, (F) HFPO-DA, (G) PFPeA, (H) diPFHxA, and (I) N4. **Notes:** developmental toxicity scores were assigned based on observed phenotypic abnormalities: normal larvae (score = 0), larvae with a single abnormality (score = 1), larvae with multiple abnormalities (score = 2), and dead larvae (score = 3). Significance relative to the negative control group was tested by the Pearson chi-square test (*p < 0.05; **p < 0.01; ***p < 0.001). NC: negative control group. Each stacked bar plot displays the distribution of developmental toxicity scores within each exposure group, with colors denoting different scores.

Other malformations were observed at a lower frequency. Curved body axes appeared after PFOS, PFOA, and N4 exposure; pericardial edema occurred with 6:2 FTS and TPS-PFBS; bent tails were rare and only associated with 6:2 FTS; short trunks were exclusively observed after PFPeA exposure. However, none of these treatment groups exhibited significant differences compared to their respective negative control groups. Representative images of the malformations are shown in Figure 2A. Full dose–response curves for all the malformations observed for each tested PFAS are available in SI Figure S2.

In addition to lethality and malformations, we also observed hatching delays across multiple PFAS treatments. Caution is warranted when interpreting data from PFAS treatments with relatively high rates of delayed hatching. The chorion, an envelope around the egg that serves as a barrier to prevent exogenous damage, may affect the penetration of chemicals during toxicity testing.^26^ Delayed hatching rates exceeding 30% were consistently observed across all PFOA treatment groups, suggesting potential underestimation of its lethality due to this barrier effect. A detailed analysis of delayed hatching is provided in SI Figure S3.

### An Integrative Scoring Approach Reveals PFAS-Induced Developmental Toxicity Trends

The overall developmental toxicity of PFAS was characterized using the combined developmental effect score, which increased with higher PFAS concentrations. To compare the relative developmental toxicity of the tested PFAS, we identified the lowest concentration at which a significant difference from the control was observed (*p* < 0.001). PFOS and PFOA exhibited strong developmental toxicity, as evidenced by significant differences across all treatment groups compared to the negative control. In contrast, none of the HFPO-DA or PFPeA treatment groups were significantly different from the NC group with a *p*-value < 0.001. The overall developmental toxicity decreased in the following order: PFOS, N4, PFOA, 6:2 FTS, TPS-PFBS, diPFHxA, K-PFBS, HFPO-DA, and PFPeA (Figure 3).

### PFAS at Environmentally Relevant Concentrations Induce Significant Transcriptional Changes in Developmental and Lipid Metabolism Pathways

Based on observed malformations and existing knowledge of PFAS toxicity, we analyzed the transcriptional changes in five genes (*fgf10a*, *igf1*, *fabp10a*, *pparg*, and *mttp*) to further contextualize how PFAS exposure disrupts developmental and metabolic pathways (Figure 4). Gene expression was analyzed at two levels of exposure: a higher compound-specific dose that induced malformations without significant lethality, and a uniform lower dose (0.03 μM) reflecting environmentally relevant concentrations.^8^

**Figure 4:**
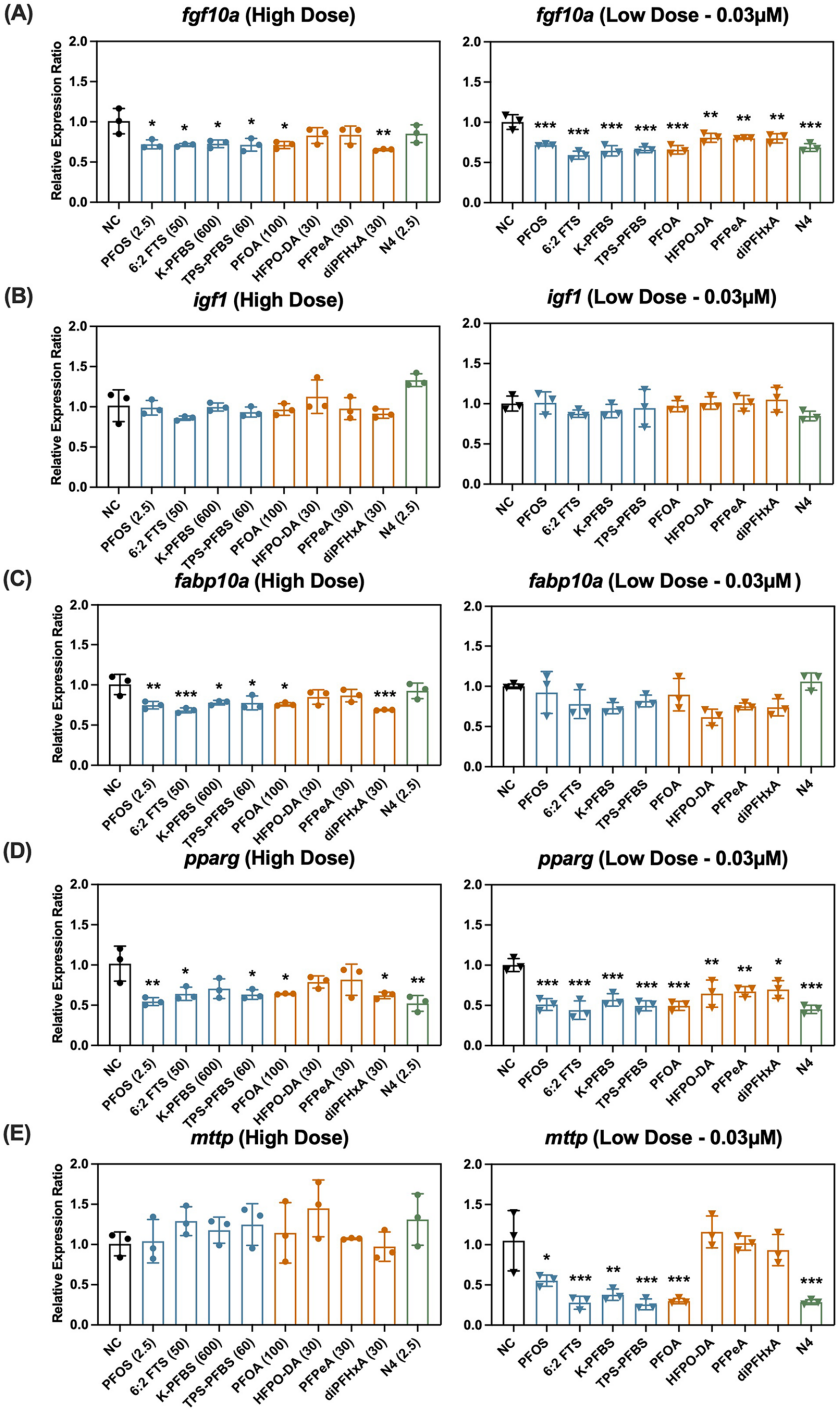
Relative expression ratios of (A) *fgf10a*, (B) *igf1*, (C) *fabp10a*, (D) *pparg*, and (E) *mttp* after PFAS exposure. **Notes**: differences in the relative expression ratio between the control and PFAS groups were determined via one-way ANOVA followed by Tukey’s multiple comparisons test (*p < 0.05; **p < 0.01; ***p < 0.001). For high-dose exposures, the PFAS concentrations (in μM) are provided following each PFAS label; the low-dose exposure (0.03 μM) was identical for all PFAS. Negative control (NC) group is in black; sulfonic acids are in blue; carboxylic acids are in orange; and perfluorosulfonamide is in green.

To investigate the potential underlying mechanisms of the observed developmental toxicity, we analyzed the expression of *fgf10a* and *igf1*, two critical growth factors involved in organogenesis and tissue growth, respectively.^27,28^ High-dose exposure to all PFAS except HFPO-DA, PFPeA, and N4 induced similar levels of significant downregulation of *fgf10a* expression (*p* < 0.05). At lower doses, PFAS with lower developmental toxicity (HFPO-DA, PFPeA, diPFHxA) caused only moderate downregulation of *fgf10a* (p < 0.01), whereas the other PFAS induced greater suppression (p < 0.001; Figure 4A). This pattern mirrored the incidence of failed bladder inflation (Figure 2B). No significant changes in *igf1* expression were observed at either dose (Figure 4B).

The yolk sac is a lipid-rich organ that serves as the primary nutrient source during early embryonic development, with proper lipid mobilization and metabolism being critical for nutrient absorption and utilization.^29^ Given that yolk sac edema was one of the most commonly observed malformations and has been linked to disrupted lipid metabolism after some bisphenol A exposure,^30^ we investigated the expression of three key genes *fabp10a*, *pparg*, and *mttp* involved in lipid metabolism after PFAS exposure.

Following high-dose exposure, *fabp10a* was significantly downregulated after exposure to 50 μM 6:2 FTS (*p* < 0.001), 30 μM diPFHxA (*p* < 0.001), 2.5 μM PFOS (*p* < 0.01), 600 μM K-PFBS (*p* < 0.05), 60 μM TPS-PFBS (*p* < 0.05), and 100 μM PFOA (*p* < 0.05), but not for other PFCAs or N4. In contrast, low-dose exposure (0.03 μM) did not significantly alter the *fabp10a* expression (Figure 4C). For *pparg* and *mttp*, a distinct dose-dependent pattern was observed, similar to that of *fgf10a*: low-dose exposure induced more significant gene expression changes than did high-dose treatment. At the lower doses, all the tested PFSAs, PFOA, and N4 consistently downregulated *pparg* and *mttp*, with TPS-PFBS, 6:2 FTS, PFOA, and N4 causing more than a 2-fold decrease. For *pparg*, high-dose treatment significantly altered its expression for most PFAS, except for K-PFBS, HFPO-DA, and PFPeA (Figure 4D). The expression of *mttp* was upregulated after high-dose PFAS exposures, though none of these upregulations were significant (Figure 4E).

## Discussion

### PFAS Lethal Potency: A Window into Acute Toxic Effects

The lethal potency trends observed in this study are consistent with established structure–activity relationships for PFAS: developmental toxicity tends to increase with chain length for PFAS within a functional group class (e.g., PFSAs);^19,31^ sulfonic acid PFAS exhibit greater lethal potency to zebrafish than carboxylic acids of similar chain lengths,^17,19,31^ and HFPO-DA, a current-use PFAS, is less potent.^32^ The LC_50_ values determined for N4 (28.78 μM), PFOS (37.97 μM), and PFOA (352.90 μM) highlight their relatively higher acute toxicity compared to other PFAS tested.

Comparisons with LC_50_ data from the EPA ECOTOX Knowledgebase (https://cfpub.epa.gov/ecotox/) revealed considerable variability across studies. PFOS and PFOA, the most frequently studied PFAS, have LC_50_ values in zebrafish embryos ranging from 2.2 to 107.6 mg/L (4.4 to 215.1 μM)^18,31,33–37^ and 41.41 to 759 mg/L (100 to 1833 μM), respectively.^18,31,37–45^ Our calculated LC_50_ values for PFOS (37.97 μM) and PFOA (146.13 μM) fall within the lower end of these ranges, indicating higher toxicity. Differences in experimental conditions (e.g., timing and duration of exposure, presence or absence of the chorion, whether media was renewed, etc.) likely contributed to this variability, emphasizing the importance of standardized zebrafish embryo assay protocols.^46^ Standardization not only enhances the reliability of toxicity data but also improves the robustness of assessments used to inform environmental and public health policies.

### PFAS-Induced Malformations: Sublethal Effects as Early Indicators of Long-Term Challenges

The occurrence of “weak” larvae across all PFAS exposures further reinforces concerns regarding the underestimation of toxicity when assessments focus solely on lethality. By incorporating sublethal end points, we capture a better understanding of adverse effects at concentrations below those inducing mortality. This is particularly relevant for regulatory frameworks, as lethality-based assessments may overlook significant developmental disruptions that could have long-term environmental and human health implications.

Our findings highlight significant sublethal effects, with failed swim bladder inflation and yolk sac edema emerging as the most prevalent developmental abnormalities. PFAS-induced failed swim bladder inflation might be related to thyroid hormone disruption,^47–49^ while yolk sac edema likely reflects lipid metabolism disruption resulting from abnormal lipid accumulation in the yolk sac.^30^ Further mechanistic studies are critical to elucidate the pathways through which PFAS exert their toxicity, as understanding these mechanisms is essential for translating developmental outcomes in zebrafish to potential risks for human health.

The calculated EC_50_ values provided insight into the toxic effects of those PFAS that did not elicit statistically significant responses in the lethality assessment, such as K-PFBS and TPS-PFBS, which are commonly used in PAGs. Notably, TPS-PFBS caused more frequent occurrences of failed swim bladder inflation and yolk sac edema compared to K-PFBS, indicating a greater toxic effect at equivalent exposure levels.

### Some Less-Studied Functional Groups Are of Concern

The analysis of developmental scores revealed a general trend in the developmental toxicity of PFAS, with PFOS, N4, and PFOA demonstrating the highest potency, while HFPO-DA and PFPeA exhibited minimal toxicity within the tested concentrations. Among the most potent compounds, PFOS and PFOA have been phased out in the United States,^3^ whereas N4 remains actively used in industry, warranting careful consideration of its potential risks. In contrast, the limited toxicity observed for HFPO-DA and PFPeA aligns with industry efforts to replace legacy PFAS with shorter-chain or branched alternatives to reduce bioaccumulation potential and toxicity.^32^ However, recent evidence suggests that some emerging PFAS may still bioaccumulate or exert chronic toxic effects.^50^

We also observed concerning toxicity associated with less-studied functional groups such as perfluorosulfonamides (i.e., N4) and dicarboxylic acids (i.e., diPFHxA). Dicarboxylic PFAS exhibited more potent developmental toxicity than the monocarboxylic PFAS of equivalent fluorinated carbon chain lengths in this study, suggesting that having two carboxylate groups may provide dual anchoring points for stronger interactions with biological targets. For example, dicarboxylic acids can enhance hydrogen bonding to developmental receptors and increase calcium ion binding,^51^ disrupting calcium signaling pathways essential for zebrafish embryonic development.^52^ These properties may prolong the bioactivity and lead to greater disruption during sensitive developmental windows. Nevertheless, limited information is available regarding the toxicological profiles of perfluoroalkyl dicarboxylic acids as a chemical group. Our finding highlights the need for further research to determine whether other perfluoroalkyl dicarboxylic acids similarly exhibit greater toxicity.

While the developmental toxicity score (and its threshold for significant differences across exposure groups by dose) provides an additional integrative measure of developmental toxicity, it may not capture the full spectrum of potential PFAS-induced effects. Further investigations incorporating additional end points and mechanistic insights are needed to gain a more comprehensive understanding of the developmental toxicity of PFAS and broaden PFAS assessments beyond traditional perfluoroalkyl acids.

### PFAS Alter the Expression of Genes Involved in Cell Growth, Organ Development, and Lipid Metabolism

The downregulation of *fgf10a* following PFAS exposure provides mechanistic insights into the observed developmental toxicity, particularly the high incidence of failed bladder inflation. *fgf10a*, a member of the fibroblast growth factor family, plays critical roles in tissue development, organogenesis, and repair (ZFIN: https://zfin.org/ZDB-GENE-030715-1#), and serves as a well-established marker of zebrafish swim bladder development.^53,54^ Its consistent suppression at lower exposure levels suggests that PFAS may disrupt *fgf10a*-mediated signaling pathways, leading to impaired bladder formation. Although the swim bladder is a fish-specific organ, *fgf10a* is evolutionarily conserved with human *FGF10*, which is essential for lung, salivary gland, and intestinal development as well as for wound healing and epithelial regeneration.^55–57^ Thus, PFAS-induced suppression of *fgf10a* in zebrafish embryos implies that potential perturbations could occur in human pulmonary or glandular tissues, particularly during sensitive developmental windows. This aligns with several epidemiological and *in vitro* studies linking prenatal PFAS exposure with altered lung function, thyroid hormone imbalance, and delayed growth trajectories—effects consistent with dysregulated *FGF* and *IGF* signaling axes.^58–61^ Future investigations examining additional genes, such as *wnt2*, *sox2*, and *fgfr2b*, could further clarify this molecular cascade and strengthen causal inference between PFAS exposure and disrupted organ development. To validate *fgf10a* and related genes as biomarkers of PFAS-induced failed swim bladder inflation and facilitate the transition from *in vivo* to *in vitro* approaches, future studies could use targeted knockdown or CRISPR-based gene editing to establish causal roles, complemented by *in situ* hybridization or immunohistochemistry to localize and confirm altered gene expression during critical developmental stages.

To investigate the potential mechanism underlying PFAS-induced yolk sac edema and its relevance to human health, we examined the expression of *igf1*, a key regulator of cardiac development.^62^ Yolk sac edema is commonly associated with cardiac dysfunction, such as abnormal heart development, reduced cardiac output, and circulation issues, which can lead to fluid accumulation around the yolk sac.^63,64^ However, *igf1* expression remained unchanged across all of the treatments, indicating it was not affected at the tested doses. Further work is needed to identify other molecular targets more directly related to PFAS-induced yolk sac edema, and to facilitate the translation of zebrafish embryo assay observations to human health contexts.

In addition to developmental pathways, PFAS-induced toxicity may also involve the disruption of lipid metabolism, as indicated by altered expression of *fabp10a*, *pparg*, and *mttp*. Among high-dose exposures, *fabp10a* was most downregulated by certain PFAS at relatively lower concentrations, including 30 μM diPFHxA, 50 μM 6:2 FTS, and 2.5 μM PFOS, suggesting their greater ability to induce lipid metabolism dysregulation. K-PFBS at 600 μM, TPS-PFBS at 60 μM, and PFOA at 100 μM all induced similar levels of *fabp10a* suppression, indicating greater potency of TPS-PFBS and PFOA relative to K-PFBS. As *fabp10a* mediates fatty acid binding and transport (ZFIN: https://zfin.org/ZDB-GENE-020318-1), its suppression suggests impaired lipid homeostasis that could hinder embryonic development; such dysregulation is also associated with metabolic disorders in humans, including obesity, diabetes, cardiovascular disease, and hepatic steatosis.^65^ The absence of significant *fabp10a* changes at the tested low dose highlights the potential to establish exposure thresholds for this type of PFAS toxicity.

Conversely, more significant suppression in *pparg*, *mttp*, and *fgf10a* expression was observed following low-dose PFAS exposure than high-dose PFAS exposure. This may suggest a nonmonotonic dose–response (NMDR) behavior, which is known to occur in response to some endocrine-disrupting chemicals.^66,67^ Specific PFAS have been established to be endocrine-disrupting chemicals,^45,47,68^ and NMDR has been reported for HFPO-DA-induced brain gene expression alterations.^69^ One possible explanation is that low doses activate specific signaling pathways or compensatory mechanisms, leading to pronounced gene regulation, while high doses overwhelm these systems or induce cellular stress responses that blunt transcriptional activity.^66,70^ However, the specific mechanisms underlying the potential NMDR observed in *pparg*, *mttp*, and *fgf10a* expression remain unclear. As these genes are involved in pathways regulated by endocrine signals (e.g., lipid metabolism and growth factors), their suppression suggests that these PFAS may act as endocrine disruptors, further amplifying developmental and metabolic toxicity.

Compared with *fabp10a*, *pparg* expression was more consistently and significantly downregulated by all PFAS at low doses. As an upstream transcription regulator of lipid metabolism, *pparg* responds to environmental cues (such as chemical exposures) and regulates the expression of multiple downstream genes, including *fabp10a*.^71^ Its early role in the signaling cascade may increase its sensitivity to PFAS-induced disruption. *mttp*, which is essential for lipid transport and lipoprotein assembly (ZFIN: https://zfin.org/ZDB-GENE-040419-2), was also notably suppressed at low doses. Consistent with our findings, previous studies have reported decreased *mttp* expression in mice following PFOS exposure.^72^ The greater than 2-fold downregulation of *mttp* by TPS-PFBS, 6:2 FTS, PFOA, and N4 highlights their potential to impair lipid transport processes and induce metabolic stress, even at environmentally relevant concentrations.

### Toward Sustainable Practices in Semiconductor Manufacturing: A Toxicological Perspective on Photolithography-Related PFAS

The semiconductor industry is facing one of its most pressing sustainability challenges: balancing chemical performance with environmental and human health. While photolithography uses of PFAS are highly controlled and constitute a relatively minor source of environmental PFAS release,^8^ it is one of the industries using a structurally diverse suite of PFAS.^3^ In this work, we tested nine legacy and emerging photolithography-relevant PFAS. Key malformations observed include impaired swim bladder inflation and yolk sac edema, with higher concentrations proving lethal to some larvae. Moreover, PFAS exposure was associated with the altered expression of genes involved in developmental and lipid metabolism pathways, even at an environmentally relevant concentration. Our findings highlight concerns for some understudied functional groups, i.e., perfluoroalkyl dicarboxylic acids and perfluorosulfonamides. Compounds such as TPS-PFBS and N4, still actively used in industry, also demonstrated adverse effects.

The semiconductor industry is actively addressing sustainability challenges, working within strict regulatory frameworks and performance requirements, to minimize environmental and health impacts. For example, the early transition from long-chain PFAS (e.g., PFOS-based PAGs) to short-chain analogues (e.g., PFBS-based formulations) was driven by evidence that shorter-chain PFAS are less bioaccumulative and persistent.^73^ This proactive shift—grounded in the best available science at the time—demonstrates the industry’s commitment to safer innovation, but gaps in available data on other PFAS can hinder progress. Our findings help guide and accelerate efforts toward developing safer alternatives by identifying specific functional groups of concern, particularly those that remain under-characterized. This allows for the redesign of molecules by removing or modifying harmful structural features and blocking metabolic pathways that generate toxic intermediates. By linking molecular behavior to biological outcomes, mechanism-driven innovation shifts focus from *post hoc* protections to inherently safer chemistries that maintain efficacy without compromising health or environmental safety.

Developing and adopting new chemistries remains challenging because semiconductor manufacturing involves thousands of tightly interdependent process steps, each with stringent performance, reliability, and safety requirements. Introducing new materials requires extensive validation to ensure compatibility without risking product integrity or safety. To better reflect real-world conditions, future work should expand this framework to encompass mixture effects, chronic effects, and environmental transformations.

## Supplementary Material



## Notes

Experimental details and methods including zebrafish husbandry and embryo collection; instrumental analysis of PFAS concentrations and surface tension; RNA extraction and cDNA synthesis for qPCR gene expression analysis; and figures supporting the results of zebrafish malformations and delayed hatching (PDF)

## Abbreviations

(PFAS): per- and polyfluoroalkyl substances
(TPS-PFBS): triphenylsulfonium perfluoro-1-butanesulfonate
(PAGs): photoacid generators
(PFPeA): perfluoropentanoic acid
(6:2 FTS): 1H,1H,2H,2H-perfluorooctane sulfonic acid
(fabs): fabrication facilities
(PFCAs): perfluoroalkyl carboxylic acids
(PFSAs): perfluoroalkyl sulfonic acids
(hpf): hour post fertilization
(N4): bis­(1,1,2,2,3,3,4,4,4-nonafluoro-1-butanesulfonyl)­imid
(NC): negative control
(PC): positive control
(PFOS): perfluorooctane sulfonic acid
(K-PFBS): potassium nonafluorobutane sulfonate
(PFOA): perfluorooctanoic acid
(HFPO–DA): undecafluoro-2-methyl-3-oxahexanoic acid
(PFPeA): perfluoropentanoic acid
(diPFHxA): octafluoroadipic acid
(ppm): parts per million
(dpf): day post fertilization
(LC_50_): lethal concentration 50%
(EC_50_): half-maximal effective concentration
(SD): standard deviation
(ANOVA): analysis of variance
(CI): confidence interval
(NMDR): nonmonotonic dose–response
