# Supplementary material for: Developmental Toxicity of Photolithography-Relevant Per- and Polyfluoroalkyl Substances (PFAS) Reveals Concerns for Less-Studied Functional Groups
Source: Environ Sci Technol. 2025 Dec 24;60(1):167–80. doi: 10.1021/acs.est.5c09577 (PMC12810226; doi:10.1021/acs.est.5c09577)
Supplement: Supplementary file 1 [file es5c09577_si_001.pdf]

Supporting Information for:

Developmental Toxicity of Photolithography-relevant Per- and Polyfluoroalkyl Substances (PFAS) Reveals Concerns for Less-studied Functional Groups

Yuexin Cao[1], Hajar Smaili[1], Hazel Q. Shanks[2], Brooke E. Tvermoes[3], Shan Niu[4], Ruiwen Chen[1], Neil A. Hukriede[2] and Carla A. Ng[1,5*]

[1] Department of Civil & Environmental Engineering, University of Pittsburgh, Pittsburgh, PA, USA, 15261

[2] Center for Integrative Organ Systems, Department of Cell Biology, University of Pittsburgh, Pittsburgh, PA, USA, 15213

[3] Chief Sustainability Office, IBM, Durham, NC, USA, 27709

[4] Advanced Interdisciplinary Institute of Environment and Ecology, Beijing Normal University, Zhuhai, Guangdong, China, 519087

[5] Department of Environmental and Occupational Health, University of Pittsburgh, Pittsburgh, PA, USA, 15261

*** Corresponding author: carla.ng@pitt.edu**

There are 2 sections, 13 pages, 3 figures and 2 tables included in the supporting information.

Section S1. Supporting experimental methods

Section S1.1 Zebrafish husbandry and embryo collection

Wild-type (AB strain) adult zebrafish (*Danio rerio*) were raised in a continuous flow-through system at 28.5 °C, pH 7-8, under a 14:10 h light:dark cycle at the zebrafish facility in the School of Medicine at the University of Pittsburgh. To obtain embryos, breeding tanks were set up overnight with 5 male and 5 female zebrafish in each tank. Glass marbles and plastic plants were added to stimulate spawning, and mesh spawn traps prevented adult zebrafish from consuming the embryos. Embryos were collected 30-60 minutes after removing the divider in the breeding tank the next morning after breeding. The fertilized embryos were selected, with coagulated embryos discarded.

Section S1.2 Instrumental analysis

The instrumental analysis was adapted from our previous study.[1] PFAS quantification was conducted following the U.S. EPA Draft Method 1633 using ultrahigh-performance liquid chromatography coupled with a triple quadrupole mass spectrometer (UHPLC-MS/MS). The analysis was performed with an electrospray ionization source operating in negative-ion mode, using a Thermo Scientific Vanquish Flex UHPLC system and TSQ Quantis mass spectrometer (Waltham, MA, USA).

For the UHPLC analysis, the mobile phase was a mixture of solvent A (DI water with 20mM ammonium acetate) and solvent B (Methanol). The mobile phase began with 10% solvent B with a total flow rate of 0.35 mL/min for 0.5 min, followed by 95% solvent B at 4 min, and was maintained at 95% for the remainder of the chromatography run (6 min).

DiPFHxA was analyzed using an Agilent N4 HILIC 50 × 2.1mm, 1.8 µm analytical column for separation without a guard column (Table S1). The mobile phase started at 95% solvent B with a total flow rate of 0.25 mL/min for 0.5 min, then decreased to 5% solvent B by 10 min, and was held constant until the end of the chromatography run (16 min). For other PFAS, separations were achieved using a Thermo Hypersil Gold C18, 50 x 2.1 mm, 1.9 µm HPLC column with a column guard and a Thermo Hypersil Gold C18, 50 x 4.6 mm, 1.9 µm HPLC column as a delay column (Table S1). MS acquisition transitions are provided in SI Table S1. MS analysis was performed in HESI negative mode with a gas temperature of 325 °C, vaporizer temperature of 300 °C, and sheath gas and auxiliary gas set to 50 and 10 Arb, respectively. Quality control criteria required that errors between measured and nominal stock concentrations remain below 10%.

Table S1. MS acquisition transitions in UHPLC-MS/MS.

Compound	Retention Time (min)	Precursor (m/z)	Product (m/z)	Collision Energy (V)
**C18 Column**				
Perfluoropentanoic acid (PFPeA)	3.7	263	68.9	18
		263	219.07	8
Perfluorobutane sulfonic acid (PFBS)	3.9	299	80	32
		299	99	27
Undecafluoro-2-methyl-3-oxahexanoic acid (HFPO-DA)	4.35	285	119	16.54
		285	169	5.38
1H,1H,2H,2H-Perfluorooctane sulfonic acid (6:2 FTS)	4.7	427.012	80.471	28
		427.012	407	21
Perfluorooctanoic acid (PFOA)	4.71	413.05	168.768	15.07
		413.05	219	12
		413.05	369.018	7.65
Bis(1,1,2,2,3,3,4,4,4-nonafluoro-1-butanesulfonyl)imid (N4)	4.8	579.8	256.4	15
		579.8	296.9	32
Perfluorooctane sulfonic acid (PFOS)	4.84	498.988	80.096	39.88
		498.988	99.014	38.32
**HILIC Column**				
Octafluoroadipic acid (diPFHxA)	13.6	289	169	12
		289	181	8

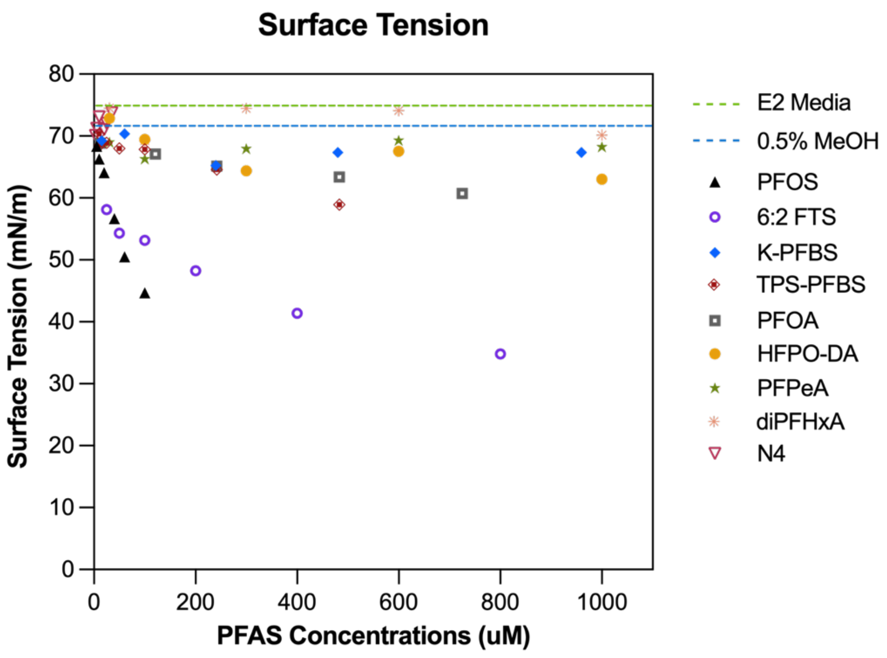

Figure S1. Surface tension of PFAS solutions.

Among the nine PFAS tested, only PFOS and 6:2 FTS showed a significant decrease in surface tension with increasing concentrations. However, despite this, no significant lethality was observed in zebrafish larvae exposed to 6:2 FTS, whereas significant lethality occurred in other PFAS exposures, such as certain treatment groups of PFOA and N4, where surface tension did not decrease notably with higher concentrations. Thus, surface tension is unlikely to be a direct factor contributing to zebrafish lethality.

Section S1.3 RNA extraction, cDNA synthesis, primer validation, qPCR conditions, and gene expression analysis

Total RNA was extracted using the RNeasy Plus Universal Mini Kit (QIAGEN) according to manufacturer's instructions. RAN concentration and purity were assessed using a NanoDrop™ 1000 Spectrophotometers (Thermo Scientific), with purity validated by an A_{260}/A_{280} ratio between 1.9 and 2.1. RNA was reverse transcribed into cDNA using the qSscript™ cDNA SuperMix (QuantaBio) based on manufacturer's instructions. Gene expression was quantified using the QuantStudio 12K Flex Real-Time PCR System (qPCR, Thermo Scientific) with Applied Biosystems™ PowerUp™ SYBR™ Green Master Mix (Fisher). Each experimental condition was tested in triplicate, with three biological replicates per condition. No template controls (NTCs) including all reaction components except sample were included to monitor for potential contamination.

b-actin1 was selected as the housekeeping gene as an internal control based on the stability analysis of candidate reference genes.[2] Gene-specific primers (*β-actin1*, *fabp10a*, *pparg*, *mttp*, *fgf10a* and *igf1*) were listed in Table S2 and obtained from IDT. Primer efficiencies were validated using ten-fold/five-fold serial dilutions of cDNA and calculated from the formula: Efficiency (%) = $(10^{-1/\text{slope}} - 1) \times 100$ (SI Table S2). The qPCR cycling conditions were as follows: 50 °C for 2 min and 95 °C for 2 min, followed by 40 cycles of denature (95 °C) for 15 s, anneal (60 °C) for 15 s and extend (72 °C) for 1 min. Melt curve analysis was performed with the following dissociation conditions: ramp rate of 1.6 °C/s at 95 °C for 15 s, 1.6 °C/s at 60 °C for 1 min, and 0.15 °C/s at 95 °C for 15 s. Relative gene expression was quantified using the $2^{-\Delta\Delta Ct}$ method, normalized to *β-actin1*.[3]

Table S2. Sequence and efficiency of primers for the tested genes.

**Gene Name (Symbol)**	**Accession Number**	**Sequences of primers (5′-3′)**	**Efficiency (%)**
actin, beta 1 (*b-actin1*)[4]	NM_131031	F: CGAGCAGGAGATGGGAACC<br>R: CAACGGAAACGCTCATTGC	99
fatty acid binding protein 10a, liver basic (*fabp10a*)[5]	NM_152960	F: TTACGCTCAGGAGAACTACG<br>R: CTTCCTGATCATGGTGGTTC	96
peroxisome proliferator-activated receptor gamma (*pparg*)	NM_131467	F: GATATGGTGGACACGCAGAC<br>R: TCTCGTAGTCGATGCCTGAT	91
microsomal triglyceride transfer protein (*mttp*)[6]	NM_212970	F: GCTTCCAGATGCCAGCCTAT<br>R: GTGACATCCACGGTTTCTGC	101
fibroblast growth factor 10a (*fgf10a*)	NM_182870	F: AACACTTACTGGAAGCACTTG<br>R: CTTGACTAAATTCGGATGGTAGG	95
insulin-like growth factor 1 (*igf1*)	NM_131825	F: TGACATTGCCCGCATCTCAT<br>R: GAGACAGCGCATGGTACACT	97

Note: Reference annotations following gene names indicate the sources of the primer sequences. For genes without annotations, primers were designed using Primer-BLAST (https://www.ncbi.nlm.nih.gov/tools/primer-blast/).

Section S2. Figures supporting the results

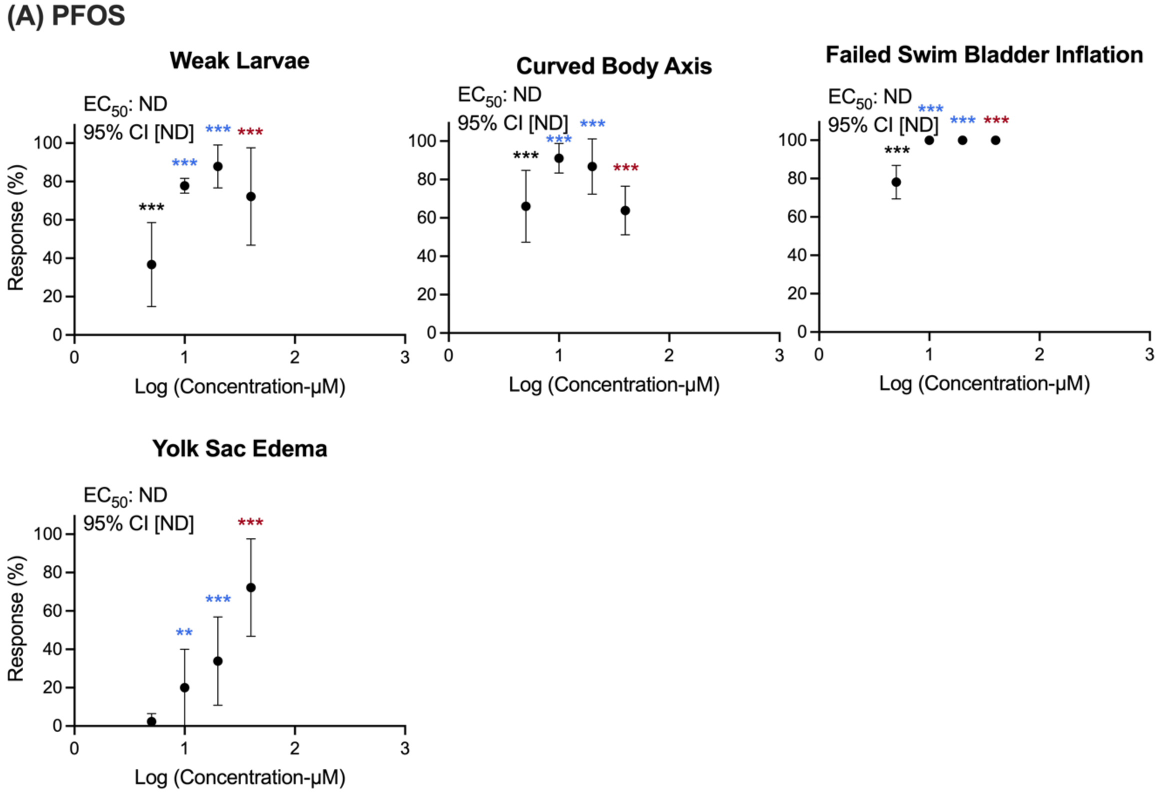

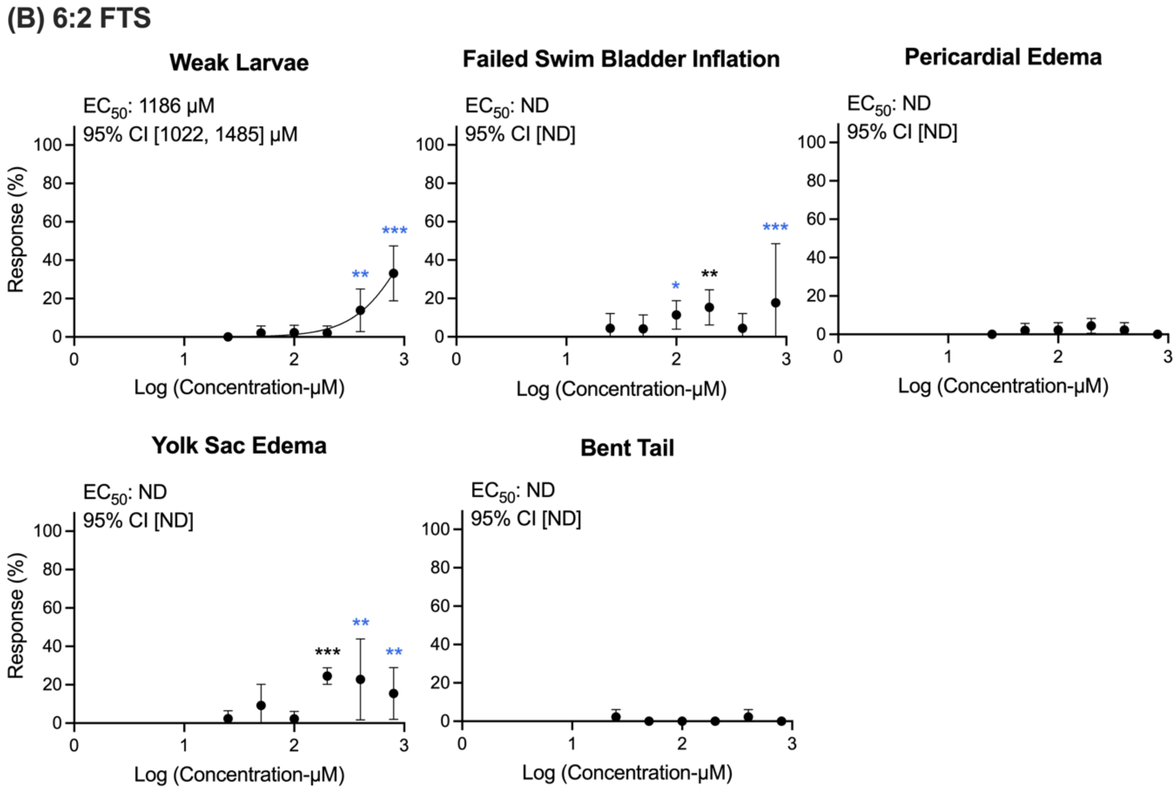

(C) K-PFBS

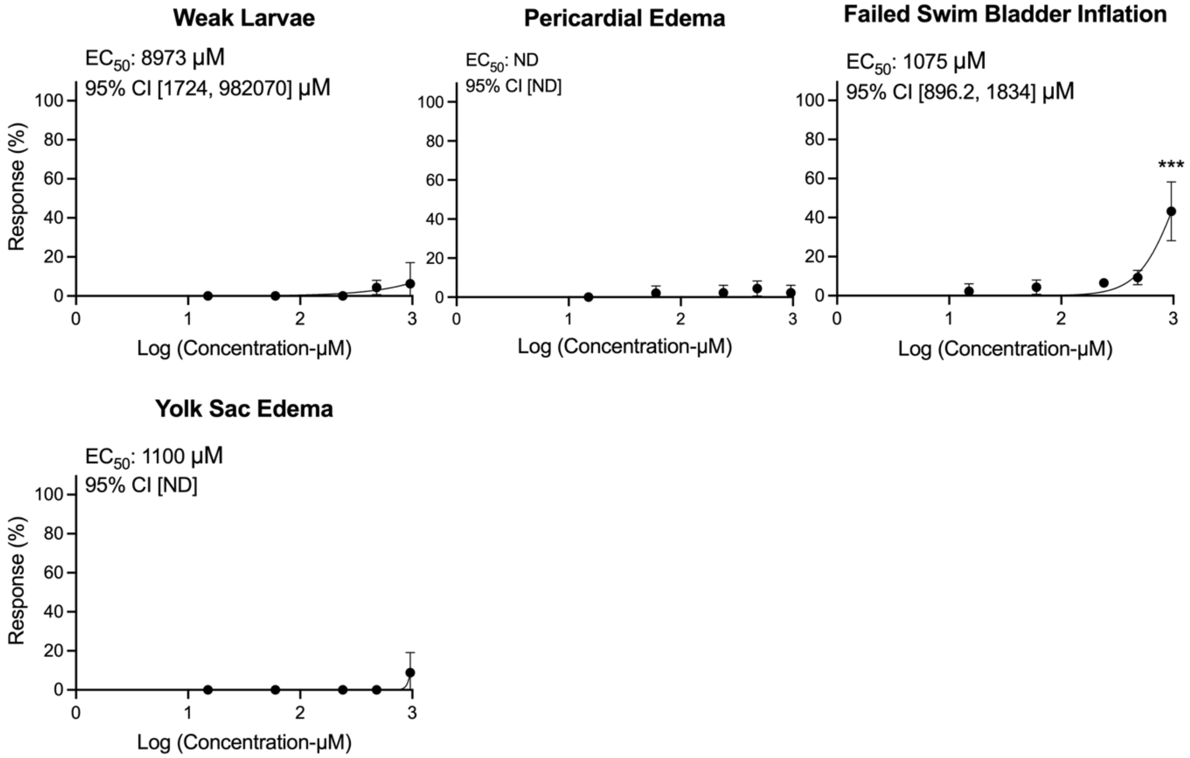

(D) TPS-PFBS

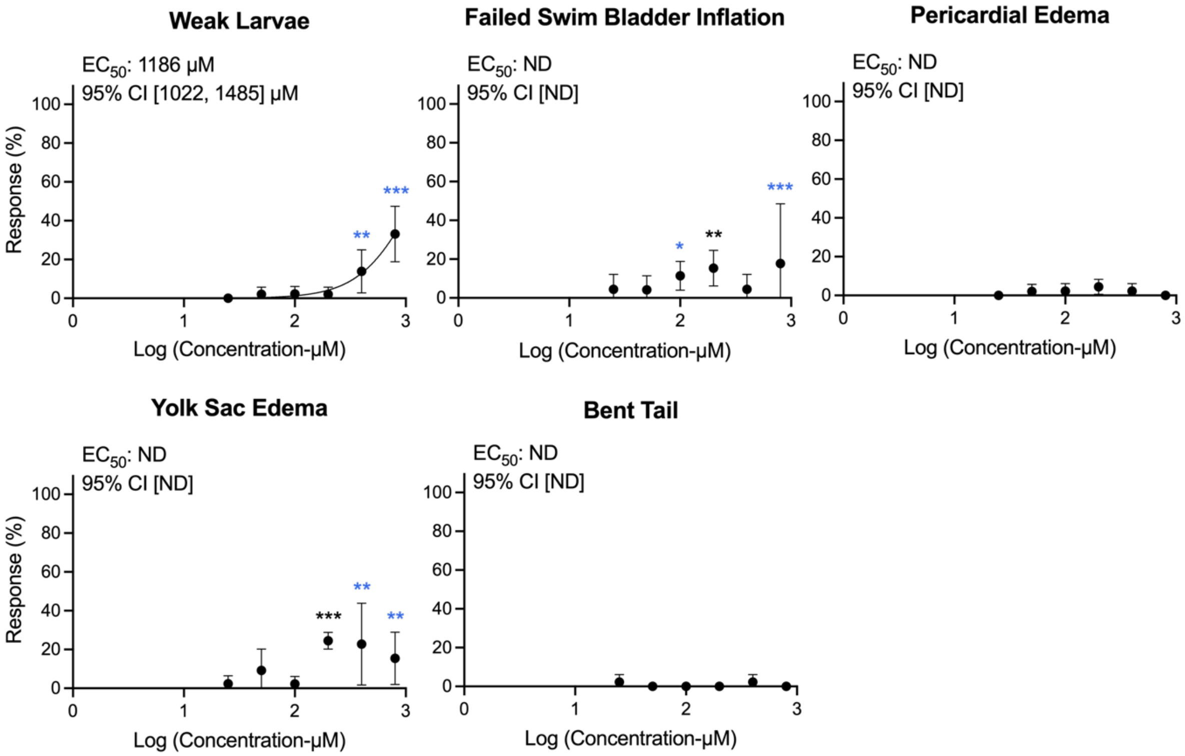

(F) HFPO-DA

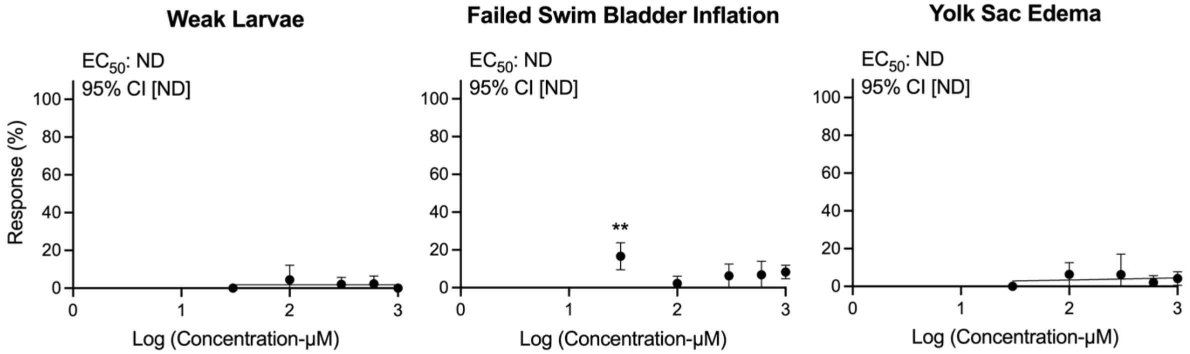

(G) PFPeA

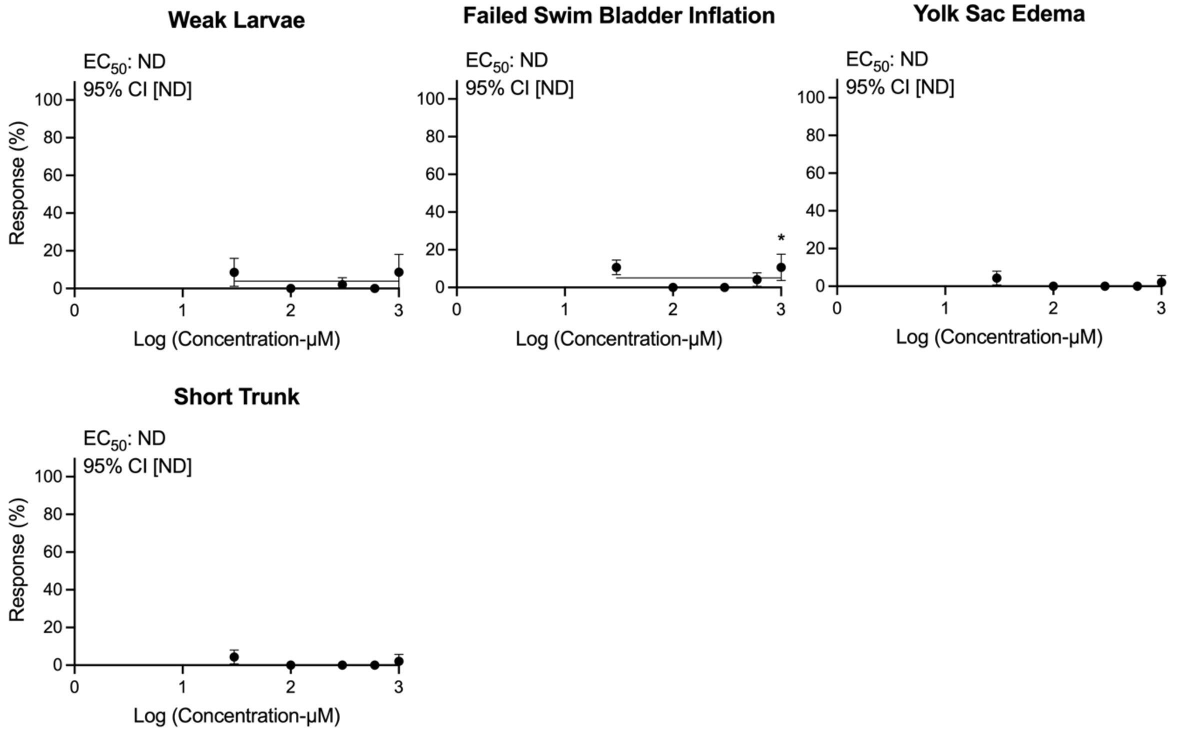

(H) diPFHxA

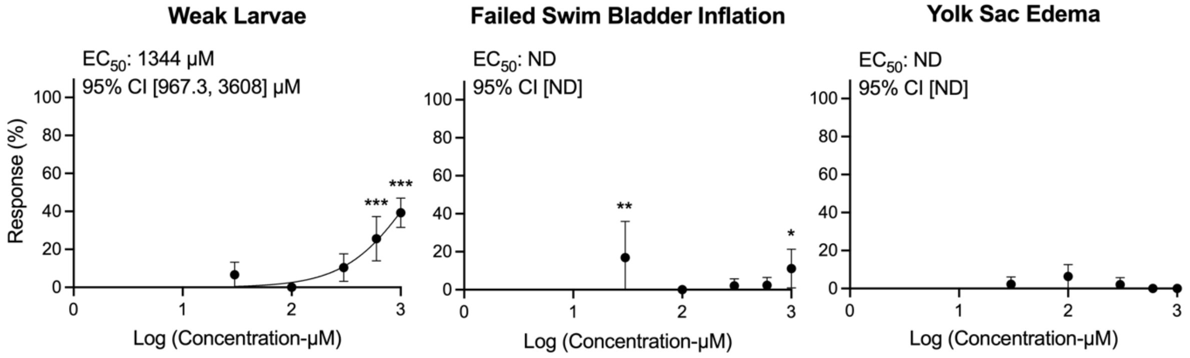

(I) N4

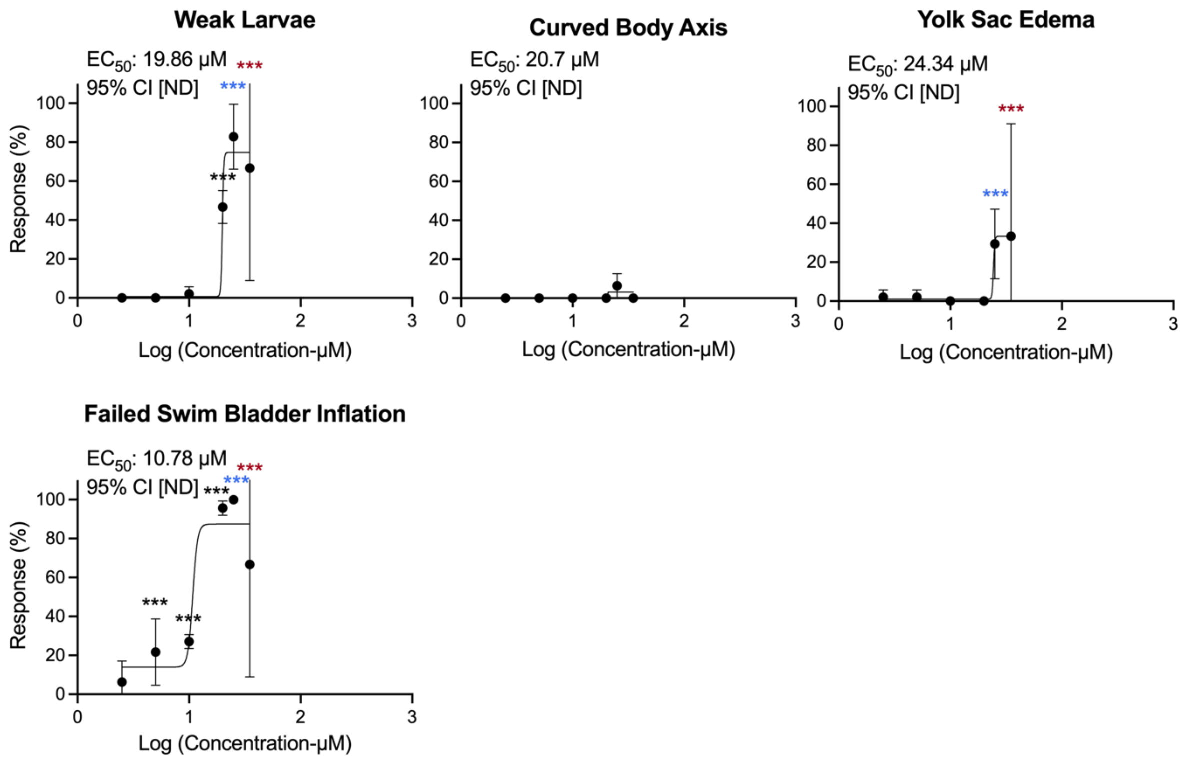

Figure S2. Dose-response curves of sublethal endpoints observed after zebrafish embryos exposed to (A) PFOS, (B) 6:2 FTS, (C) K-PFBS, (D) TPS-PFBS, (E) PFOA, (F) HFPO-DA, (G) PFPeA, (H) diPFHxA and (I) N4. **Notes**: statistical significance relative to the negative control group was determined by the Pearson chi-square test/Fisher exact test (* $p < 0.05$; ** $p < 0.01$; *** $p < 0.001$). Blue and red stars indicate that the statistical analysis result was from a set of data including more than 10% and 40% missing data representatively. "ND" indicates that the tested concentrations did not result in a sufficient number of sublethal outcomes to determine the EC_{50} value accurately or 95% confidence interval (CI) data cannot be calculated based on the current data due to high variability. Error bar indicates one standard deviation (SD) from the mean.

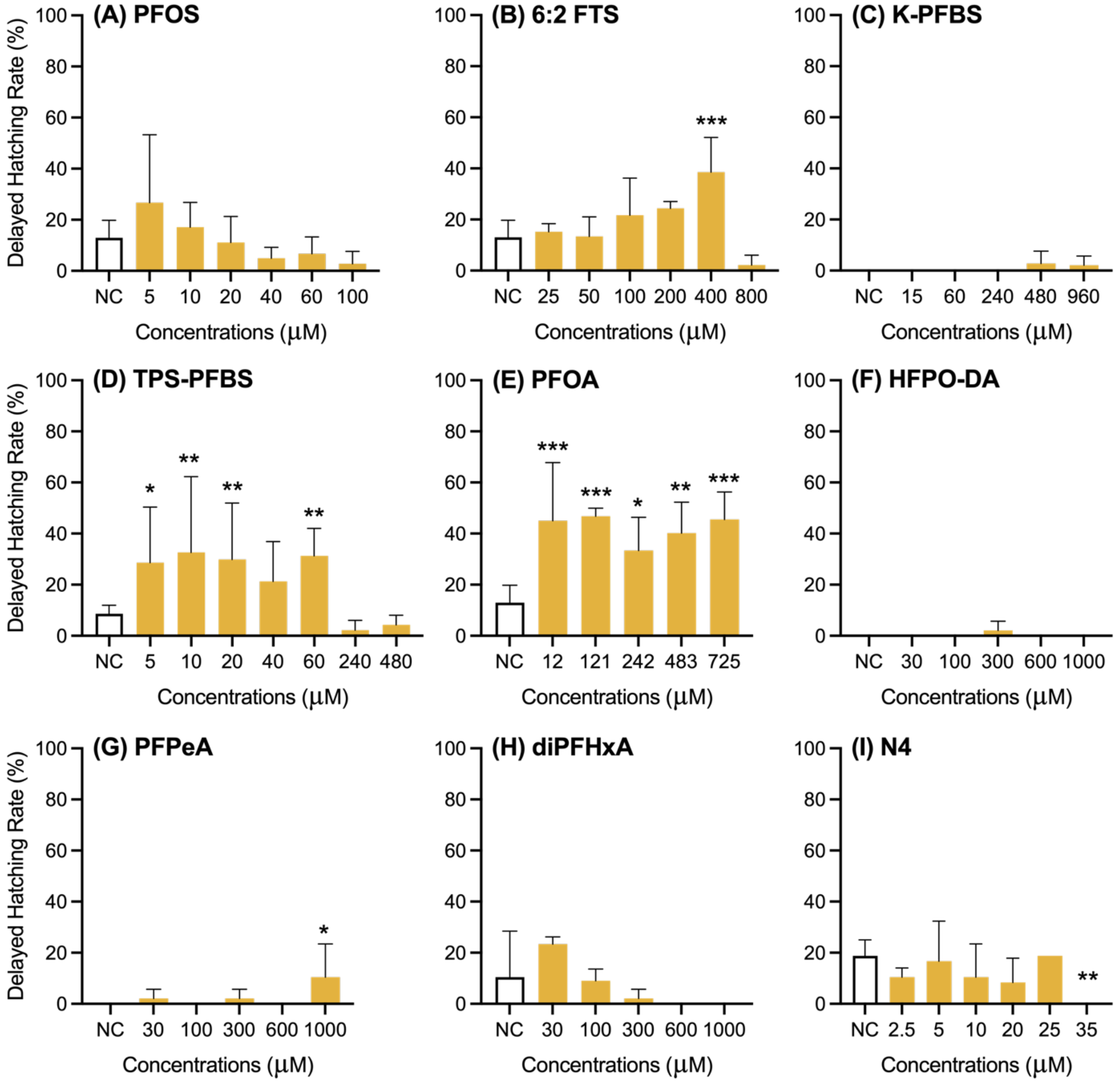

Figure S3. Zebrafish embryo delayed hatching rate following exposure to (A) PFOS, (B) 6:2 FTS. (C) K-PFBS, (D) TPS-PFBS, (E) PFOA, (F) HFPO-DA, (G) PFPeA, (H) diPFHxA and (I) N4. **Notes**: the NC is represented in white, while treatment groups are shown in orange. Statistical significance relative to the negative control group was assessed by the Pearson chi-square test/Fisher exact test (* $p < 0.05$; ** $p < 0.01$; *** $p < 0.001$). Error bar indicates the SD.

We did not observe delayed hatching in zebrafish embryos exposed to K-PFBS, HFPO-DA or most lower concentrations of PFPeA. In contrast, delayed hatching was detected in both the treatment and negative control groups for PFOS, 6:2 FTS, TPS-PFBS, PFOA, diPFHxA and N4. Increasing concentrations of PFOS and diPFHxA were associated with a decreasing delayed

hatching rate; however, there were no statistically significant differences between the treatment and control groups. Notably, TPS-PFBS and PFOA exposure resulted in a significantly higher delayed hatching rate, with all PFOA treatment groups exhibiting delayed hatching rates exceeding 30%, which was significantly different from the negative control group (Figure S3).

In the developmental toxicity assay, the hatching status revealed distinct patterns regarding delayed hatching in zebrafish embryos exposed to various PFAS. K-PFBS, HFPO-DA, and most lower concentrations of PFPeA did not exert a significant effect on the hatching process, suggesting lower toxicity in terms of their developmental impact compared to other tested PFAS. This observation aligns with previous findings that shorter-chain PFAS, such as K-PFBS and HFPO-DA, exhibit reduced lethality and developmental disruption. In contrast, exposure to PFOS, 6:2 FTS, TPS-PFBS, PFOA, diPFHxA, and N4 led to delayed hatching, consistent with studies indicating that PFOA[7] and PFOS[8] have the propensity to cause delayed hatching. The delayed hatching of zebrafish embryo might be related to disruptions in hatching gland enzymes and embryo movement, both of which are critical for digesting the chorion.[7] However, the underlying mechanism of delayed hatching from PFAS exposure is still unclear. It is also challenging to discern whether the delayed hatching observed is directly attributable to chemical exposure or to individual variability, as we observed some delayed hatching in the negative control groups as well. The timing of hatching within a population typically varies over a range of about one day, which adds complexity to the interpretation of delayed hatching data.[7]

We did not observe a clear trend correlating PFAS concentration or chain length with delayed hatching rates. Interestingly, as PFOS and diPFHxA concentrations increased, the delayed hatching rate decreased, although this difference was not statistically significant when compared to the control group. This might be because of the lower surfactant properties of PFOS and diPFHxA; at higher concentrations, PFAS could reduce surface tension, potentially facilitating hatching. However, this pattern was not consistent across all tested PFAS.

References

(1) Niu, S.; Zhu, X.; Chen, R.; Winchell, A.; Gao, P.; Barchowsky, A.; Buchanich, J. M.; Ng, C. Personal Wearable Sampler for Per- and Polyfluoroalkyl Substances Exposure Assessment. *Environ. Sci. Technol. Lett.* **2024**, *11* (4), 301–307. https://doi.org/10.1021/acs.estlett.4c00026.

(2) Khazaee, M.; Guardian, M. G. E.; Aga, D. S.; Ng, C. A. Impacts of Sex and Exposure Duration on Gene Expression in Zebrafish Following Perfluorooctane Sulfonate Exposure. *Environ Toxicol Chem* **2020**, *39* (2), 437–449. https://doi.org/10.1002/etc.4628.

(3) Pfaffl, M. W. A New Mathematical Model for Relative Quantification in Real-Time RT–PCR. *Nucleic Acids Research* **2001**, *29* (9), e45. https://doi.org/10.1093/nar/29.9.e45.

(4) Jantzen, C. E.; Annunziato, K. M.; Cooper, K. R. Behavioral, Morphometric, and Gene Expression Effects in Adult Zebrafish (Danio Rerio) Embryonically Exposed to PFOA, PFOS, and PFNA. *Aquat Toxicol* **2016**, *180*, 123–130. https://doi.org/10.1016/j.aquatox.2016.09.011.
(5) Venkatachalam, A. B.; Lall, S. P.; Denovan-Wright, E. M.; Wright, J. M. Tissue-Specific Differential Induction of Duplicated Fatty Acid-Binding Protein Genes by the Peroxisome Proliferator, Clofibrate, in Zebrafish (Danio Rerio). *BMC Evol Biol* **2012**, *12*, 112. https://doi.org/10.1186/1471-2148-12-112.
(6) Mentec, H. L. Impact de perturbateurs endocriniens sur la stéatose hépatique et sa progression pathologique.
(7) Hagenaars, A.; Vergauwen, L.; De Coen, W.; Knapen, D. Structure–Activity Relationship Assessment of Four Perfluorinated Chemicals Using a Prolonged Zebrafish Early Life Stage Test. *Chemosphere* **2011**, *82* (5), 764–772. https://doi.org/10.1016/j.chemosphere.2010.10.076.
(8) Shi, X.; Du, Y.; Lam, P. K. S.; Wu, R. S. S.; Zhou, B. Developmental Toxicity and Alteration of Gene Expression in Zebrafish Embryos Exposed to PFOS. *Toxicology and Applied Pharmacology* **2008**, *230* (1), 23–32. https://doi.org/10.1016/j.taap.2008.01.043.
